# Antimicrobial Effects of Thymosin Beta-4 and Ciprofloxacin Adjunctive Therapy in *Pseudomonas aeruginosa* Induced Keratitis

**DOI:** 10.3390/ijms21186840

**Published:** 2020-09-18

**Authors:** Thomas W. Carion, Abdul Shukkur Ebrahim, Spandana Alluri, Thanzeela Ebrahim, Tressa Parker, Julia Burns, Gabriel Sosne, Elizabeth A. Berger

**Affiliations:** Department of Ophthalmology, Visual & Anatomical Sciences, Wayne State University School of Medicine, Detroit, MI 48201, USA; tcarion@med.wayne.edu (T.W.C.); eabdulsh@med.wayne.edu (A.S.E.); spandana.alluri@med.wayne.edu (S.A.); thanzeela24@gmail.com (T.E.); trparker20@gmail.com (T.P.); juliaburns2001@gmail.com (J.B.); gsosne@med.wayne.edu (G.S.)

**Keywords:** cornea, infection, bactericidal, antibiotics, antimicrobial peptides, *Pseudomonas aeruginosa*

## Abstract

Prior work has indicated that thymosin beta 4 (Tβ4) administered with ciprofloxacin markedly improves disease outcome for *Pseudomonas aeruginosa* (PA)-induced keratitis. As a result, the goal of the current study was to elucidate mechanisms by which Tβ4 mitigates the corneal response; specifically, regarding its bactericidal influence and potential synergy with ciprofloxacin. An in vitro approach was carried out using minimum inhibitory concentration (MIC) assays to assess bactericidal activity against PA. In addition, antimicrobial peptide (AMP) production was evaluated at the mRNA levels using human corneal epithelial cells in response to lipopolysaccharide (LPS) challenge. The results of the MIC assays did not show direct bactericidal activity with Tβ4 alone, although ciprofloxacin exhibited significant killing at concentrations far lower than clinically dosed. Tβ4, however, displayed an indirect effect on bacterial killing, as shown by an upregulation of AMPs and related molecules. The cumulative data from this study indicate an indirect bactericidal role of Tβ4, as well as a synergistic relationship with ciprofloxacin. Furthermore, ciprofloxacin alone was found to influence cellular functions that otherwise have yet to be reported. These results highlight a mechanism of intracellular communication for Tβ4 and further strengthen its development as an adjunct therapy with antibiotics for corneal infections.

## 1. Introduction

Bacterial keratitis is a deleterious infection of the cornea that causes corneal scarring, opacification, and perforation. While this disease is commonly associated with contact lens wear, risk factors also include refractive corneal surgery and immunocompromised conditions [1,2]. Current standard of care consists of topical antibiotics with steroids; however, it is important to note the associated risks and potential side effects that necessitate safer, more effective therapies. Antibiotic treatment is compounded by antibiotic resistance and can cause toxicity issues that compromise visual outcome [3,4]. Although the use of relevant corticosteroids effectively dampen the host inflammatory response, steroid-induced glaucoma, impaired wound healing, and increased risk of infection are among the considerable drawbacks [5]. To this end, this study is focused on the development of an alternative therapy for the treatment of infectious corneal diseases.

Thymosin beta 4 (Tβ4) was first isolated and identified from calf thymus 30 years ago, by Goldstein et al. [6]. This 43 amino acid protein is highly conserved across species and is the most ubiquitously expressed peptide of the β-thymosins, expressed in all tissues and cell types except red blood cells with concentrations ranging from 1 × 10^−5^ to 5.6 × 10^−1^ M [7]. Initially described as an actin sequestering protein, Tβ4 has been more recently found to play a significant role in wound healing and regenerative pathways [8,9]. Prior work using a mouse model of *Pseudomonas aeruginosa* (PA)-induced bacterial keratitis has shown that when used as an adjunct to ciprofloxacin, disease outcome was significantly improved [10]. Additionally, this combination therapy was found to downregulate key inflammatory mediators, which have been attributed to host-induced damage such as edema and corneal scarring. While it is known that ciprofloxacin directly inhibits bacterial growth, the role of Tβ4 in disease outcome is not yet fully understood. Tang et al. indicated a direct antimicrobial effect for Tβ4 that was isolated from human platelets [11], however these findings have been difficult to reproduce in the eye. Work carried out in our in vivo model revealed, after infection, decreased bacterial load in corneas treated with only antibiotic as compared to no detectable bacteria in corneas treated with the adjunct therapy [10]. These findings led us to determine the synergistic bactericidal effects of Tβ4 with ciprofloxacin in the current study.

We also assessed the indirect influence of Tβ4 on bacterial killing via the modulation of the host response. Antimicrobial peptides (AMPs) are small positively charged molecules that function to defend against invading pathogens. Human ocular surface epithelia secrete peptides from the defensin and cathelicidin families, which are thought to exert protective broad-spectrum activity against a sundry of microorganisms. AMP production is also enhanced through the activation of pro-resolution circuits [12]. Therefore, this study further assessed the impact of Tβ4 on select AMPs and pro-resolving factors.

Overall, our objective was to elucidate the mechanism(s) by which Tβ4 affects bacterial killing, host response, and ciprofloxacin activity in the setting of bacterial keratitis. Our findings indicate that Tβ4 does in fact play an indirect role in bacterial killing, while synergistically enhancing the inhibitory effects of antibiotics.

## 2. Methods

### 2.1. Human Corneal Epithelial Cell Culture

Human telomerase-immortalized corneal epithelial cells (HUCLs), kindly provided by the laboratory of Fu-Shin Yu, were used for the AMP studies. These cells, previously infected with a retroviral vector encoding human telomerase reverse transcriptase to generate an immortalized cell line, have been characterized and validated as a viable model for corneal epithelial cell investigation [13,14]. The HUCLs were placed in keratinocyte-serum-free medium (K-SFM) supplemented with growth factors (EGF and bovine pituitary extract, Invitrogen—Life Technologies, Carlsbad, CA, USA) and were maintained in a humidified 5% CO_2_ incubator, at 37 °C. Before treatment, cells were seeded at a density of 0.2 × 10^6^/mL (total volume of 2 mL) into 6-well culture plates that were precoated with fibronectin collagen (FNC Coating Mix, Athena Environmental Service, Inc., Baltimore, MD, USA) and cultured in defined keratinocyte SFM for 2 days. Upon confluency, cells were treated as described below. HUCLs were used between passages 3–5 for all experiments.

### 2.2. Minimum Inhibitory Concentration (MIC) Assay

Minimum inhibitory concentration (MIC) assays were executed using a 96-well microdilution technique using the following four treatment groups: media only (control), Tβ4, ciprofloxacin, and Tβ4 + ciprofloxacin. Serial dilutions were carried out in PTSB with a final total volume of 200 µL/well. Final concentrations of Tβ4 ranged from 0.8 to 0.0015%; ciprofloxacin ranged from 0.3 to 0.0006%. For the Tβ4 + ciprofloxacin combination treatment group, Tβ4 was used at a final concentration of 0.1% and varying concentrations of ciprofloxacin ranged from 0.3 to 0.0006%.

Bacterial inoculum was prepared, as previously described [10]. Each well was inoculated with a 5 µL aliquot of PA ATCC 19,660 containing either 10^5^ or 10^6^ CFU/mL, then, incubated for 18–20 h in 5% CO_2_, at 37 °C. Optical density (OD) readings were collected by spectrophotometry. Results are reported as OD_600_ ± SEM.

### 2.3. Measurement of Antimicrobial Peptides (AMPs)

Cultured HUCLs were divided into the following four treatment groups: media (control), Tβ4 (0.1%), ciprofloxacin (0.3%), and Tβ4 + ciprofloxacin. Dosing of both Tβ4 and ciprofloxacin have been previously established [10]; further 0.3% ciprofloxacin is the current standard of care for the treatment of bacterial keratitis. All four groups were stimulated with 25 μg/mL lipopolysaccharide (LPS) (PA serotype 10-derived LPS, Sigma-Aldrich, St. Louis, MO, USA) for up to 24 h. At both 6 and 24 h, total RNA was isolated from each group in triplicate, as previously described [10], using RNA-STAT 60 (Tel-Test, Friendswood, TX, USA), according to the manufacturer’s recommendations and quantified by spectrophotometric determination (260 nm). Then, a cDNA template was constructed by reverse transcribing 100 ng of total RNA, then amplified using SYBR^®^ Green Master Mix (Thermo Fisher Scientific, Waltham, MA, USA), per the manufacturer’s instruction, with the reaction mixture previously described [15]. All primers were generated using Primer3 PCR v. 4.1.0 primer design software and real-time RT-PCR was carried out using the CFX Connect Real-Time RT-PCR Detection System (BioRad, Hercules, CA, USA). The relative standard curve method was used to establish levels of transcript expression normalized to β-actin [16]. Results are reported as the mean fold change ± SD as compared with untreated controls.

### 2.4. Statistics

All assays were carried out from a minimum of three independent experiments with representative data from a typical experiment shown. Data are presented as mean + SD unless indicated otherwise. Results were obtained in a blinded fashion and analyzed by a one-way ANOVA followed by Bonferroni’s multiple comparison test (GraphPad Prism, San Diego, CA, USA) with data considered significant at *p* < 0.05.

## 3. Results

### 3.1. Tβ4 Does Not Appear to Directly Influence Bactericidal Activity

**Figure 1:**
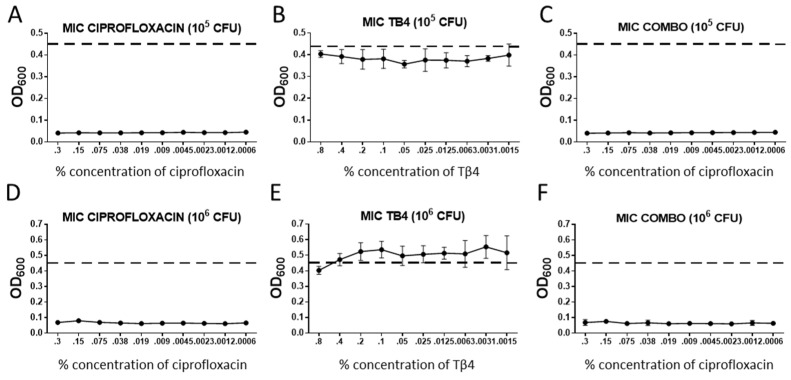
Results from MIC assays represented as OD_600_ readings + SD for (**A**,**D**) ciprofloxacin; (**B**,**E**) thymosin beta 4 (Tβ4); (**C**,**F**) combination Tβ4 + ciprofloxacin, measured at varying concentrations. Each group was run for a minimum inhibitory concentration based on 10^5^ (**A**–**C**) and 10^6^ (**D**–**F**) CFU/mL *Pseudomonas aeruginosa* (PA). Concentrations are denoted along the *x*-axis. For the combination treatment, cipro concentrations are along the *x*-axis and Tβ4 was used at 0.1%. Positive controls are denoted by the dashed line. *n* = 6/group/timepoint.

Bacterial killing for each treatment group was assessed using a MIC assay, the results of which are shown in Figure 1. When compared to untreated controls (dotted line), ciprofloxacin exhibited significant bactericidal activity for all concentrations of antibiotic (0.3–0.0006%) as compared with controls. Ciprofloxacin also appeared similarly effective against 10^5^ as compared with 10^6^ CFU/mL of bacteria. In contrast, all concentrations of Tβ4 remained similar to untreated controls for both concentrations of bacteria, thus, indicating a lack of bactericidal effect. Combination treatment showed similar trends to that observed for ciprofloxacin alone at both concentrations of bacteria.

### 3.2. Tβ4 Upregulates AMP Expression in Human Telomerase-Immortalized Corneal Epithelial Cells HUCLs

**Figure 2:**
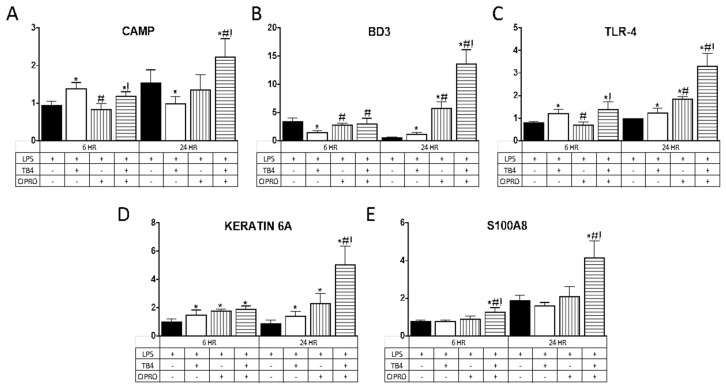
mRNA expression of (**A**) CAMP; (**B**) BD3; (**C**) TLR4; (**D**) keratin 6A; and (**E**) S100A8 in human telomerase-immortalized corneal epithelial cells (HUCLs) at 6 and 24 h, following LPS stimulation (25 µg/mL). Tβ4 and ciprofloxacin were used at a concentration of 0.1% and 0.3%, respectively. Results are reported as relative fold change of the gene of interest, normalized to β-actin ± SD. *n* = 3/group/timepoint. * *p* < 0.05 vs. LPS, # *p* < 0.05 vs. Tβ4, ! *p* < 0.05 vs. ciprofloxacin.

AMP expression was evaluated as an indirect mechanism by which adjunctive Tβ4 therapy reduces bacterial load, contributing to improved disease outcome in *Pseudomonas* keratitis. Select AMPs and related molecules (Table 1) were chosen given their known expression and role in corneal epithelial cells. As illustrated in Figure 2, Tβ4 alone significantly upregulated mRNA expression of CAMP at 6 h and BD3 at 24 h, whereas TLR4 and keratin 6A were elevated at both time points following LPS stimulation. There were no differences in expression levels of S100A8 at either time point following Tβ4 treatment. Ciprofloxacin alone had no influence on CAMP or S100A8 expression, whereas BD3 and TLR4 transcripts were elevated at 24 h and keratin 6A was enhanced at both time points. The combination treatment of Tβ4 + ciprofloxacin resulted in a significant upregulation of all molecules at both 6 and 24 h after LPS stimulation, with the exception of BD3 (no change at 6 h).

### 3.3. Tβ4 Influences Specialized Pro-Resolving Molecules (SPM)-Related Enzymes and Receptors in HUCLs

**Figure 3:**
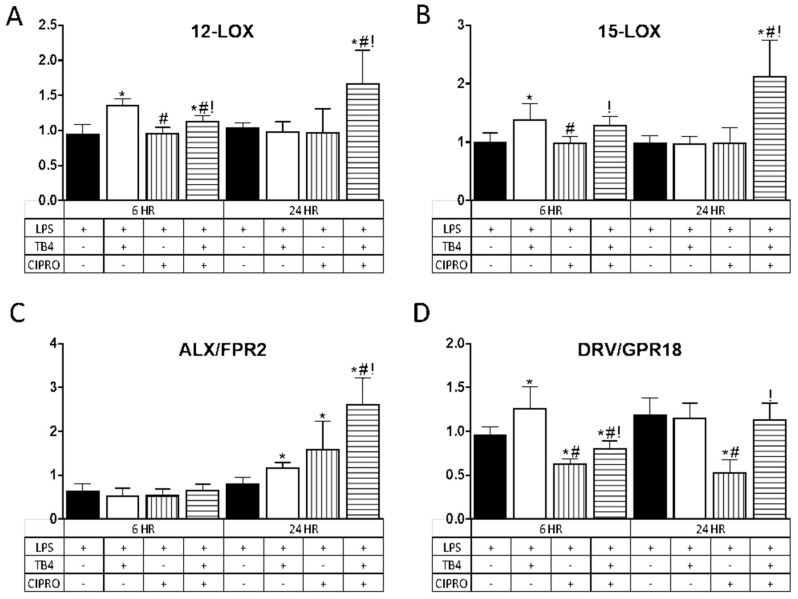
Transcript levels of select lipoxygenase enzymes (**A**) 12-LOX; (**B**) 15-LOX and specialized pro-resolving molecule (SPM) receptors (**C**) ALX/FPR2; (**D**) DRV/GPR18) in HUCLs for 6 and 24 h, after LPS stimulation (25 µg/mL) ± Tβ4 (0.1%) and ciprofloxacin (0.3%). Results are reported as relative fold change of the gene of interest, normalized to β-actin ± SD. *n* = 3/group/timepoint. * *p* < 0.05 vs. LPS, # *p* < 0.05 vs. Tβ4, ! *p* < 0.05 vs. ciprofloxacin.

In further exploring the role of adjunct Tβ4 therapy, the impact on pro-resolution pathways was examined. Results are shown in Figure 3 for two lipoxygenase enzymes, 12-LOX (A) and 15-LOX (B), known to play a prominent role in the production of specialized pro-resolving molecules (SPMs), and two SPM receptors, ALX/FPR2 (C) and DRV2/GPR18 (D). Results for the enzymes (A,B) revealed that Tβ4 induced a significant upregulation in transcript levels. at 6 h. following LPS stimulation as compared with controls; whereas the combination treatment showed a significant increase in expression at both 6 and 24 h. In contrast, transcript levels remained unchanged after treatment with ciprofloxacin only at both time points.

Regarding the receptors, ALX/FPR2 mRNA (C) remained unchanged for all treatment groups at 6 h after LPS stimulation, yet was significantly elevated at 24 h after Tβ4, ciprofloxacin, and combination treatments. DRV2/GPR18 expression (D) was elevated at 6 h following Tβ4 treatment, but significantly decreased in cells that received ciprofloxacin and the combination treatment. In fact, while DRV2/GPR18 expression was restored with the combination treatment at 24 h, this receptor remained significantly downregulated after ciprofloxacin only treatment as compared to all three groups (controls, Tβ4, and combination).

## 4. Discussion

Bacterial keratitis is an extremely debilitating and rapidly progressive infection of the cornea which can advance into sight-threatening consequences including endophthalmitis and corneal opacification. Although the criticality of treating this condition is evident, our current standard of care for bacterial keratitis topical antibiotics (ciprofloxacin, moxifloxacin, and fortified gentamicin) with judicious use of topical steroids (loteprednol and prednisolone) comes with a number of drawbacks that include antibiotic resistance, toxicity issues, steroid induced glaucoma, and increased risk for infection [3,4,5]. Furthermore, the role of corticosteroids in the adjunctive treatment of ocular infections is not well supported by evidence-based reports [5]. Consequently, it is evident that a therapeutic regimen is required that optimizes both bacterial killing and host recovery, while minimizing adverse effects and augmenting better visual outcomes. Following indications of improved disease response in corneas treated with Tβ4 as an adjunct to antibiotics, the current study further elucidated the mechanistic role of Tβ4 and its potential synergy with ciprofloxacin when used as a therapeutic for bacterial keratitis.

While it has been suggested that Tβ4 exhibits bactericidal activity, results, herein, indicate that Tβ4 does not appear to play a direct or synergistic role with antibiotic in bacterial killing. These results were somewhat surprising in light of previous work, including a report that Tβ4 showed moderate bactericidal activity against PA, *Staphylococcus aureus,* and *Staphylococcus epidermis*, although this effect was inhibited in the presence of NaCl and tears [32]. In addition, Tβ4 released and isolated from human platelets have demonstrated antimicrobial properties, although this effect could have been be influenced by an acidic pH [11]. However, our findings do corroborate results obtained from in vivo studies where there has been no indication of direct bactericidal activity regarding this molecule [10].

Next, we investigated the impact of Tβ4 on bacterial killing through indirect mechanisms, specifically the production of AMPs and related molecules in human corneal epithelial cells. Cell membrane receptors that bind Tβ4 remain largely unknown, although work carried out by Zetter has revealed an extracellular pathway for Tβ4-induced regenerative properties that involved purinergic signaling [9]. In addition, Sosne et al. reported an intercellular mechanism where Tβ4 directly targeted the NF-κB subunit RelA/p65, inhibiting translocation and DNA binding activity for pro-inflammatory gene expression [33,34]. Tβ4 has also been shown to inhibit gene promoter activation [33]. As such, we focused on changes at the mRNA level to begin exploring a potential role for this molecule to enhance intracellular communication as a transcription factor for AMP expression. Upregulation was most evident in TLR4 and keratin 6A, two key molecules in the corneal response to pathogens. TLR4, which binds LPS, is critical to ocular immune defense and resistance against PA in the infected cornea [24,25]. Keratin 6A is processed into several antimicrobial peptides in corneal epithelial cells. In fact, it has been shown that knockdown of keratin 6A significantly increased PA adherence to the intact corneal surface [23]. CAMP defends against invading pathogens and amplifies the host response through promotion of anti-inflammatory mediators [17]. BD3, a member of the family of defensins, is a cationic peptide with broad spectrum antimicrobial activity and contributes to inflammation and the innate immune response, whereby BD2 and BD3 have been shown to promote resistance against *Pseudomonas*-induced ocular infection [21]. S100A8 possesses antimicrobial activity and activates the inflammatory response [19]. The significant upregulation of all tested AMPs with the combination therapy indicates a synergistic relationship between Tβ4 and ciprofloxacin, further establishing its efficacy as a combination therapy for the treatment of bacterial keratitis.

**Figure 4:**
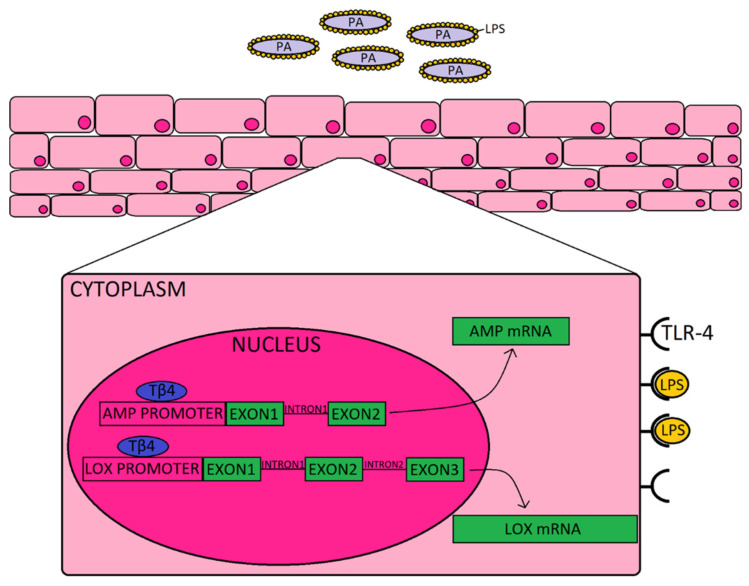
Schematic representation of how Tβ4, as an adjunct to ciprofloxacin may be enhancing the corneal epithelial cell response to *Pseudomonas* (purple) and LPS (yellow) binding to TLR4. It is proposed that Tβ4 (blue) may act as a transcription factor that is shown to bind to the promoter region, as indicated by the generalized representation shown for both AMPs (and related molecules) and lipoxygenase enzymes.

To complement the AMPs and related molecules, we also explored the impact of Tβ4 on pro-resolution enzymes, 12-LOX and 15-LOX. Prior work carried out by our lab and others has established the significance of lipoxygenase activity and activation of SPM pathways in promoting resolution and tissue restoration during ocular infection [15,29]. Activation of SPMs helps to dampen the inflammatory response and improve restoration of tissue homeostasis, and stimulates mucosal production of bactericidal peptides [12]. Previous work from our lab has shown that Tβ4 + ciprofloxacin significantly upregulated 12-LOX, an enzyme associated with the production of SPMs, and 12/15-LOX, a key marker of activated epithelial and mucosal pro-resolving pathways, in corneas of PA-infected mice [10]. The current study revealed that Tβ4 and ciprofloxacin have a synergistic effect on lipoxygenase enzymes expressed by human corneal epithelial cells in particular; whereas there does not appear to be much of an influence over SPM receptors, ALX/FPR2 and DRV2/GPR18. These summative results indicate that improved disease response observed in corneas treated with both Tβ4 and cipro may stem from their synergistic augmentation of both AMP production and activation of pro-resolution circuits that further enhance bactericidal effects, as depicted in the schematic shown in Figure 4. Although we propose, in the schematic, that extracellular Tβ4 may be internalized and translocated into the nucleus, future work is required to uncover the exact mechanism(s) by which this peptide influences gene expression.

While a synergistic relationship between Tβ4 and ciprofloxacin was expected, our findings regarding ciprofloxacin alone were particularly notable. Significant bacterial killing was detected at all tested concentrations, even those that were markedly lower than what is currently used in the clinical setting (0.0006% vs. 0.3%). These results suggest the potential for lower dosing of antibiotics, which may help to alleviate toxicity issues and the development of antibiotic resistance. Furthermore, it has been historically accepted that antibiotics are predominantly limited to bactericidal and bacteriostatic activity. Our findings suggest off-target effects on the host response, as indicated by an upregulation in AMP transcript levels. Additionally, it was observed that ciprofloxacin may have a regulatory role on SPM receptors as indicated by decreased expression of DRV2/GPR18 in the human corneal epithelial cells. These findings establish a basis for further studies on the functional activities of ciprofloxacin, as well as other antibiotics that are widely used in the clinical setting.

In light of these findings, Tβ4 shows promise as an adjunctive therapy to ciprofloxacin, in the context of corneal infections. By helping to establish its regulatory role in AMP production and activation of SPM pathways, the current work has further explicated mechanisms by which Tβ4 communicates intracellularly to improve disease outcomes as an adjunct therapy. This novel combination treatment provides an alternative to topical steroid use, and may alleviate the drawbacks associated with current standard of care.

## Figures and Tables

**Table 1:** Select antimicrobial peptides (AMPs) and related molecules.

Molecule	Background	Function
LL-37/CAMP [17,18]	LL-37/CAMP is a 37 amino acid AMP that is produced in many cells including macrophages, secondary neutrophils, NK cells, and select epithelial cells.	LL-37/CAMP has been shown to have many functions including antimicrobial activity and as a chemoattractant. It also possesses anti-inflammatory properties, modulates TLRs, and regulates IFN.
S100A8/A9 [19,20]	S100A8/A9 are Ca^2+^ binding proteins that are generally present in their heterodimeric form, which is called calprotectin. These AMPs are constitutively present in PMNs and monocytes and are heavily upregulated during trauma and inflammation.	The active release of S100A8/A9 during inflammation helps induce cytokine release and promote leukocyte recruitment. S100 proteins are often involved in the regulation of cell cycle progression and differentiation. When S100A8 complexes with S100A14, they work to regulate myeloid cell function by binding to TLR4.
hBD-1/-2/-3 [21,22]	Defensins are 2–6 kDa, cationic, microbicidal AMPs containing three pairs of intramolecular disulfide bonds.	Human beta defensins (hBDs) often work synergistically to promote bactericidal and bacteriostatic activity during infection. Specifically, they promote chemotaxis, activation, and degranulation of mast cells during inflammation.
Keratin 6A [23]	Keratin 6A codes for a 564 amino acid, 60 kDa AMP. It is a type II cytokeratin, one of a number of isoforms of keratin 6 encoded by separate genes from the gene cluster on human chromosome 12q.	Epidermis-specific keratin is involved in wound healing and shown to have antimicrobial properties. It is the main antimicrobial factor in the eye. It is involved in the activation of follicular keratinocytes after wounding, although it does not play a major role in keratinocyte proliferation or migration.
TLR4 [24,25]	TLR4 is a 95 kDa transmembrane protein of the Toll-like receptor family.	They recognize PAMPs on microbes and activates the NF-κB pathway of inflammation and the innate immune response. Cooperates with LY96 and CD14 to mediate the innate immune response to gram negative bacterial lipopolysaccharide (LPS).
12-LOX [26,27]	12-LOX is a 75 kDa enzyme composed of 663 amino acids. This protein is a lipoxygenase-type enzyme that is coded by the ALOX12 gene on chromosome 17p13.3.	Regulation of 12-LOX principally relies on the availability of its polyunsaturated fatty acids (PUFAs), which are released from lipid membranes during inflammation. This enzyme participates in arachidonic acid metabolism which can result in the activation of specialized pro-resolving molecules (SPMs).
15-LOX [28,29]	15-LOX is a 75 kDa enzyme composed of 662 amino acids. This protein is a lipoxygenase-type enzyme that is coded by the ALOX15 gene on chromosome 17p13.3.	15-LOX functions to metabolize PUFAs into SPMs that facilitate inflammation resolution.
ALX/FPR2 [30]	The ALX/FPR2 receptor is a 351 amino acid receptor that is coded for by the FPR2 gene located on chromosome 19q.13.3.	FPR2 is G protein-coupled cell surface receptor. One of the primary functions of FPR2 is its binding of SPMs, which help mediate a pro-resolving response.
DRV2/GPR18 [31]	The DRV2/GPR18 receptor is a 38 kDa protein composed of 331 amino acids. This receptor is coded for by the GPR18 gene located on chromosome 13q32.3.	GPR18 is a G protein-coupled receptor for endogenous lipid neurotransmitters. Resolvin D2, a DHA-derived SPM, is also an activating ligand for the GPR18 receptor.
